# Novel Brain Arteriovenous Malformation Mouse Models for Type 1 Hereditary Hemorrhagic Telangiectasia

**DOI:** 10.1371/journal.pone.0088511

**Published:** 2014-02-10

**Authors:** Eun-Jung Choi, Wanqiu Chen, Kristine Jun, Helen M. Arthur, William L. Young, Hua Su

**Affiliations:** 1 Center for Cerebrovascular Research, Department of Anesthesia and Perioperative Care, University of California San Francisco, San Francisco, California, United States of America; 2 Institute of Genetic Medicine, International Centre for Life, Newcastle University, Newcastle, United Kingdom; 3 Department of Neurological Surgery, University of California San Francisco, San Francisco, California, United States of America; 4 Department of Neurology, University of California San Francisco, San Francisco, California, United States of America; Odense University hospital, Denmark

## Abstract

Endoglin (ENG) is a causative gene of type 1 hereditary hemorrhagic telangiectasia (HHT1). HHT1 patients have a higher prevalence of brain arteriovenous malformation (AVM) than the general population and patients with other HHT subtypes. The pathogenesis of brain AVM in HHT1 patients is currently unknown and no specific medical therapy is available to treat patients. Proper animal models are crucial for identifying the underlying mechanisms for brain AVM development and for testing new therapies. However, creating HHT1 brain AVM models has been quite challenging because of difficulties related to deleting *Eng*-floxed sequence in *Eng^2fl/2fl^* mice. To create an HHT1 brain AVM mouse model, we used several Cre transgenic mouse lines to delete *Eng* in different cell-types in *Eng^2fl/2fl^* mice: *R26*CreER (all cell types after tamoxifen treatment), *SM22α*-Cre (smooth muscle and endothelial cell) and *LysM*-Cre (lysozyme M-positive macrophage). An adeno-associated viral vector expressing vascular endothelial growth factor (AAV-VEGF) was injected into the brain to induce focal angiogenesis. We found that *SM22α*-Cre-mediated *Eng* deletion in the embryo caused AVMs in the postnatal brain, spinal cord, and intestines. Induction of *Eng* deletion in adult mice using *R26*CreER plus local VEGF stimulation induced the brain AVM phenotype. In both models, *Eng*-null endothelial cells were detected in the brain AVM lesions, and formed mosaicism with wildtype endothelial cells. However, *LysM*-Cre-mediated *Eng* deletion in the embryo did not cause AVM in the postnatal brain even after VEGF stimulation. In this study, we report two novel HHT1 brain AVM models that mimic many phenotypes of human brain AVM and can thus be used for studying brain AVM pathogenesis and testing new therapies. Further, our data indicate that macrophage *Eng* deletion is insufficient and that endothelial *Eng* homozygous deletion is required for HHT1 brain AVM development.

## Introduction

Hereditary hemorrhagic telangiectasia (HHT), also known as Osler-Weber-Rendu (OWR) syndrome, is the first identified human disease caused by defects in a transforming growth factor-ß (TGF-ß) superfamily receptor [1]. It is an autosomal dominant genetic disorder of the blood vessels that manifests epistaxis, mucocutaneous telangiectases, and arteriovenous malformations (AVMs) in multiple organs including the brain, lung, liver, gastrointestinal tract, and spinal cord [2]. Most of HHT cases are categorized into either HHT type 1 (HHT1) caused by mutations in endoglin (*ENG*), a TGF-ß type III auxiliary receptor mapped on chromosome 9 [1], or HHT type 2 (HHT2) caused by mutations in activin receptor-like kinase 1 (*ACVRL1*; *ALK1*), a TGF-ß type I serine-threonine kinase receptor located in chromosome 12 [3]. The prevalence of brain AVM in HHT1 patients is 1000-fold higher, and in HHT2, 100-fold higher, than the prevalence in the general population (10/100,000) [4].

Brain AVM is a tangle of abnormal vessels called nidus, where blood directly shunts from arteries into veins without passing the capillary bed. These abnormal vessels tend to rupture, causing spontaneous and recurrent hemorrhages in the brain, especially in children and young adults [5]. The life-threatening intracranial hemorrhage (ICH) risk rate in HHT patients ranges from 1.4% to 2% per year [6]. Currently available therapies, including microsurgery, embolization and/or radiosurgery, are invasive and associated with considerable side effects [7], [8]. No specific medical therapy is available for brain AVM patients.

The pathogenesis of HHT brain AVMs remains largely unknown. A reliable animal model is crucial for studying disease mechanisms and testing new therapies. We and others have reported HHT2 developmental [9], [10] and adult onset brain AVM phenotypes [11] in *Alk1*-conditional knockout mice. No compelling HHT1 brain AVM model has been reported.

We have previously shown that microscopic (capillary level) cerebrovascular dysplasia can be induced by focal VEGF stimulation in adult *Eng*-haploinsufficient (*Eng^+/−^*) mice [12]. Arteriovenous (A–V) shunting has not been identified in this model. To create an adult onset HHT1 brain AVM model that closely resembles human disease, we attempted using the same strategy that we previously used to develop an HHT2 model: stereotactic injection of a cre-expressing adenoviral vector (Ad-Cre) [11] and an adeno-associated viral vector expressing vascular endothelial growth factor (AAV-VEGF). However, Ad-Cre-mediated *Eng* deletion in the brain of *Eng^2fl/2fl^* mice was very limited, in only about 1% of cells [13]. Only a few macroscopic level dysplasia vessels developed. The objective of this study is to establish HHT1 brain AVM mouse models that closely mimic the human brain AVM phenotype. The secondary objective is to explore *Eng* deletion in which cell-type is required for brain AVM development.

We used several cre transgenic lines in this study to achieve effective *Eng* deletion in *Eng^2fl/2fl^* mice. Striking brain AVM phenotypes have been developed in *Eng^2fl/2fl^;SM22α*-Cre mice spontaneously and *Eng^2fl/2fl^;R26*CreER mice after tamoxifen (TM) treatment and focal VEGF stimulation. The AVM phenotypes in these models mimic many characteristics of human brain AVM, such as arteriovenous shunting, macrophage infiltration, and microhemorrhage. Although effective *Eng* deletion in macrophages was achieved, brain AVMs did not develop in *Eng^2fl/2fl^;LysM*Cre mice after VEGF stimulation, indicating that deletion of *Eng* in macrophages is not sufficient to cause AVM formation. However, homozygous *Eng* deletion in endothelial cells might be required for brain AVM development, because *Eng*-null endothelial cells were detected in AVM vessels in both *Eng^2fl/2fl^;SM22α*Cre and *Eng^2fl/2fl^;R26*CreER mice.

## Materials and Methods

### Ethics statement

The protocol and experimental procedures for using laboratory animals were approved by the Institutional Animal Care and Use Committee (IACUC) of the University of California, San Francisco (UCSF). Animal husbandry was provided by the staff of the IACUC of UCSF, under the guidance of supervisors who are certified Animal Technologists, and by the staff of the Animal Core Facility. Veterinary care was provided by IACUC faculty members and veterinary residents located on the San Francisco General Hospital campus. All mice were housed in a pathogen-free area in 421×316 cm^2^ cages with 12 hours light/dark cycle.

### Animals

To establish various *Eng*-conditional knockout mouse lines, *Eng^2fl/2fl^* (exons 5 and 6 flanked by loxP sites) [14] mice were crossbred with *R26*CreER, *SM22α*-Cre, and *LysM*-Cre [15] mice (Jackson Laboratory, Bar Harbor, Maine).

### Viral vector stereotactic injection and TM intraperitoneal administration

AAV-VEGF or AAV-LacZ (vector control) [16] was injected into the brain of 8 to10-week-old *Eng^2fl/2fl^*;*R26*CreER and *Eng^2fl/2fl^*;*LysM*-Cre mice (6 mice per group), as previously described [17]. Briefly, mice were anesthetized with isoflurane inhalation and positioned in a stereotactic frame (David Kopf Instruments). A hole was made in the pericranium, 1 mm posterior to the coronal suture and 2 mm lateral to the sagittal suture. AAV-VEGF viral suspension (2×10^9^ genome copies) was stereotactically injected into the right basal ganglia at 3 mm in depth from the cortex. TM dissolved in corn oil (2.5 mg/25 g of body weight; Sigma-Aldrich) was injected intraperitoneally [9] to *Eng^2fl/2fl^*;*R26*CreER mice for 3 consecutive days starting from the day of viral vector injection (Figure S1A).

### Systemic latex vascular casting

Systemic latex dye perfusion, described previously [9], [11], was performed by injecting blue latex dye (Connecticut Valley Biological Supply) into the left ventricle of the heart. Brain, lung, kidney, liver, intestine and ear were collected and fixed in 4% paraformaldehyde overnight and imaged under a light microscope. Organs were then sequentially dehydrated in a methanol series, cleared in organic solvent (benzyl benzoate/benzyl alcohol, 1∶1; Sigma-Aldrich), and imaged again under a light microscope.

### Dysplasia index quantification

Two 20 µm-thick coronal brain sections per animal, 0.5 mm rostral and 0.5 mm caudal to the virus injection site, were stained with lectin (1∶200; Vector Laboratories). Three areas (to the right and left of and below the injection site) within the angiogenic region of each section were imaged under the 20X microscopic objective lens for quantification. Dysplasia index (number of vessels larger than 15 µm in diameter per 100 blood vessels) was calculated by three blinded investigators using NIH Image 1.63 software as previously described [11]–[13], [17].

### Immunohistochemistry

Mice were anesthetized with isoflurane inhalation and perfused with heparin/PBS to remove blood. Brain samples were harvested and frozen in dry ice, then sectioned into 20 µm sections. The location of AVM lesions was first identified through staining 1 of every 10 sections with lectin (1∶200; Vector Laboratories). Sections containing dysplastic vessels were then co-stained with primary antibodies against CD31 (1∶50; Abcam) and CD68 (1∶50; AbD Serotec). Each protein was subsequently visualized using fluorescently labeled secondary antibodies: Alexa Fluor 488 goat anti-rabbit IgG (1∶500; Invitrogen) for CD31, and Alexa Fluor 594 goat anti-rat IgG (1∶500; Invitrogen) for CD68. ENG expression was identified by an antibody against ENG/CD105 (1∶50; BD Pharmingen) and visualized by a biotin-conjugated secondary antibody (anti-rat IgG, 1∶500; Vector Laboratories), using the standard ABC method (Vector immunodetection kit; Vector Laboratories). Prussian blue staining was performed according to the protocol provided by the company (Iron Stain Kit; Sigma-Aldrich).

### Monocyte/macrophage isolation and culture

Monocytes/macrophages were isolated and cultured as previously described [17]. Briefly, bone marrow was collected from tibias and femurs of 8-week-old *Eng^2fl/2fl^* and *Eng^2fl/2fl^*;*LysM-*Cre mice and cultured in a macrophage-enriched medium containing mouse macrophage-colony stimulating factor (M-CSF, 7.5 ng/ml; Akron Biotech) and 10% fetal bovine serum (FBS) for 7 days.

### Real-time quantitative genomic DNA PCR analysis

DNA was isolated from the brain of 3 control *Eng^2fl/2fl^* and 3 TM-treated *Eng^2lf/2fl^*;*R26-*CreER mice, and macrophages were isolated from 3 *Eng^2fl/2fl^* and 3 *Eng^2fl/2fl^*;*LysM-*Cre mice. Recombination of the *Eng* 2fl (floxed) allele was quantified using Mx3000P QPCR System (Agilent Technologies). The gene deletion efficiency was calculated as the amount of *Eng* 2fl in experimental samples/Eng2fl levels in controls times 100. The primers used for real-time qPCR are listed in Table S1.

### Statistical analysis

Data are represented as mean ± SD. Student's t-test was used to determine a statistical significance between groups. A p value of<0.05 was considered statistically significant. The survival curve of Eng2fl/2fl;SM22α-Cre mice was made and the difference between male and female mice was analyzed using Prism 6 software.

## Results

### Developmental onset of brain AVM in *Eng^2fl/2fl^*;*SM22α*-Cre mice

**Figure 1:**
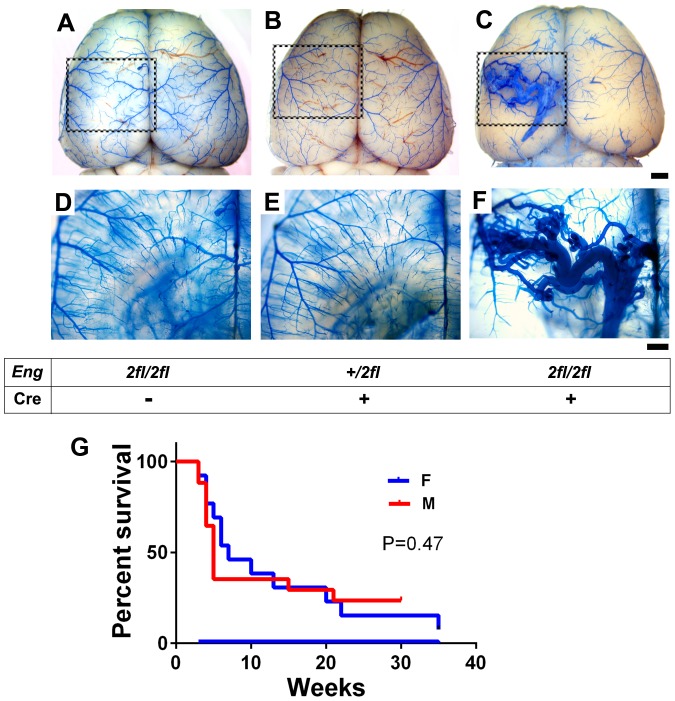
Developmental onset AVM in the postnatal brain of *Eng^2fl/2fl^*;*SM22α*-Cre mice. Representative images of latex dye casting show the cerebrovasculature in the brain of 5-week-old (A) *Eng^2fl/2fl^*, (B) *Eng^+/2fl^*;*SM22α*-Cre, and (C) *Eng^2fl/2fl^*;*SM22α*-Cre mice. D–F: Enlarged images of dotted boxes shown in A–C after the brain tissue was cleared in organic solvent. The latex-perfused vasculature inside the brain can be observed. Tangled and dilated vessels were detected only in the brain of *Eng^2fl/2fl^*;*SM22α*-Cre mice. Scale bars: 1 mm in A–C and 500 µm in D–F. G: Survival curve of *Eng^2fl/2fl^*;*SM22α*-Cre mice. There was no difference between male (M) and female (F) mice (P = 0.47).

The *SM22α*-Cre transgenic mouse induced target gene deletion in vascular smooth muscle cells during the embryonic developmental stage. In addition to vascular smooth muscle cells, *SM22α*-Cre transgene has been shown to mediate gene deletion in endothelial cells and some other cell-types [10]. To test if *SM22α*-Cre-mediated *Eng* deletion in the embryo leads to postnatal brain AVM, we crossbred *Eng^2fl/2fl^* mice with *SM22α*-Cre mice. Three different mouse groups were obtained: *Eng^2fl/2fl^*, *Eng^+/2fl^*;*SM22α*-Cre, and *Eng^2fl/2fl^*;*SM22α*-Cre. The AVM phenotype (greatly enlarged and tortuous vessels) was detected in the brain of 90% (18 out of 20) of 5-week-old *Eng^2fl/2fl^*;*SM22α*-Cre mice that had *Eng* homozygous deletion in Cre-expressing cells (Figure 1C and 1F). More than 80% of the mice had one lesion, while the remaining 20% had 2–3 lesions. No AVM phenotype was observed in the brain of *Eng^2fl/2fl^* littermates that did not have Cre transgene (wildtype) (Figure 1A and 1D) and *Eng^+/2fl^*;*SM22α*-Cre mice that had one *Eng* allele deletion (heterozygous, Figure 1B and 1E). AVMs were also found in the spinal cord (Figure S2C) and intestine (Figure S2F) of *Eng^2fl/2fl^*;*SM22α*-Cre mice, but not in *Eng^2fl/2fl^* and *Eng^+/2fl^*;*SM22α-*Cre mice (Figure S2A, S2B, S2D, and S2E). The brain lesions varied in size, location (Figure S3A and S3B), and number. There was latex dye found in the veins (indicative of A–V shunting), and hemorrhage in some brain and spinal cord lesions (Figure S3C and S3D). More than 50% of the *Eng^2fl/2fl^*;*SM22α*-Cre mice died before 6 weeks of age (Figure 1G). The survival rates were similar in males and females (P = 0.47).

### Adult onset brain AVM in *Eng^2fl/2fl^;R26*CreER mice after TM treatment and focal angiogenic stimulation

**Figure 2:**
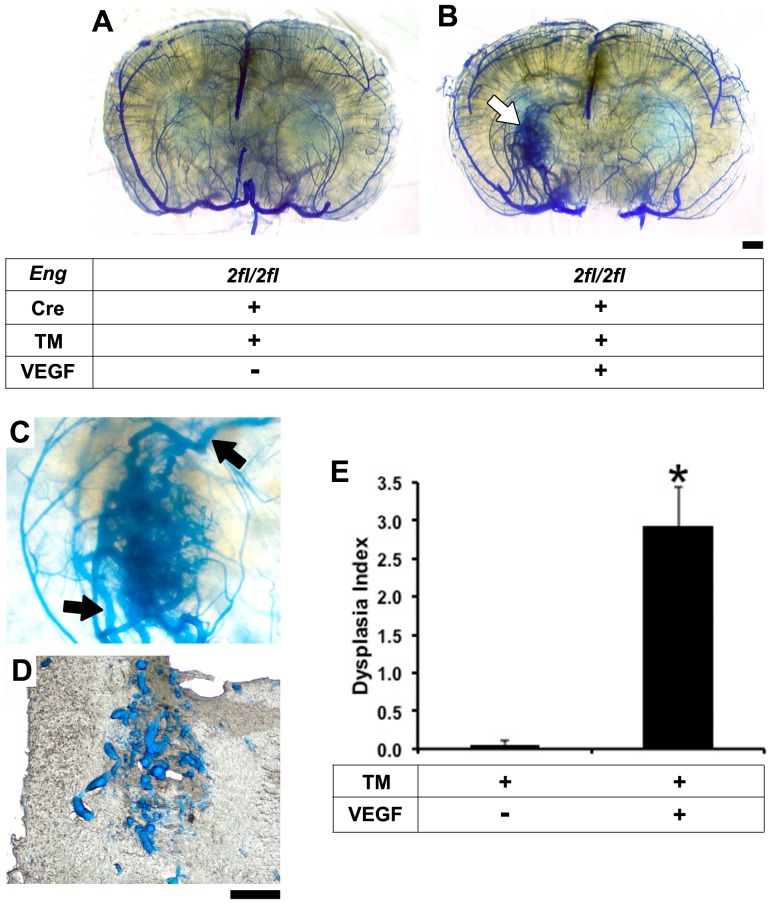
Adult onset brain AVM in *Eng^2fl/2fl^;R26*CreER mice after TM and VEGF treatment. A: Coronal view of latex-perfused adult *Eng^2fl/2fl^*;*R26*CreER brain showing no AVM phenotype 8 weeks after TM treatment. B: AVM phenotype (white arrow) developed in the brain of *Eng^2fl/2fl^*;*R26*CreER mice 8 weeks after intrabrain injection of AAV-VEGF and intraperitoneal injection of TM. C: An enlarged image of the AVM lesion. Latex-perfused veins are clearly shown at the top and bottom of the lesion (black arrows). D: Enlarged vessels observed in a 50 µm frozen section that cuts through the AVM lesion shown in C. E: Quantification of dysplasia index. Data: mean ± SD. *: *p*<0.05. n = 6 per group. Scale bars: 1 mm in A and B and 500 µm in C and D.

To induce the adult onset HHT1 brain AVM phenotype, we utilized the *R26*CreER transgenic mouse in which Cre expression is activated by TM treatment [9] to conditionally delete the *Eng* gene. *Eng* deletion in adult *Eng^2fl/2fl^*;*R26*CreER mice was induced by intraperitoneal injection of TM for 3 consecutive days (Figure S1A). About 60% of *Eng*-floxed allele was deleted in the brain (Figure S1B). *Eng* deletion alone did not affect the established vasculature in the adult brain (Figure 2A). However, the AVM phenotype developed in AAV-VEGF-induced brain angiogenic foci 8 weeks after induction of *Eng* deletion and angiogenesis (Figure 2B). Notably, the lesions mimicked the human brain AVM nidus (Figure 2C) and consisted of markedly enlarged vessels (Figure 2D). More enlarged vessels were detected in AAV-VEGF-injected sites (angiogenic foci) than in AAV-LacZ-injected (control vector) sites in the brain of TM-treated *Eng^2fl/2fl^*;*R26*CreER mice (dysplasia index: 2.9±0.5 versus 0.05±0.05, *p*<0.05, Figure 2E). Since the brain AVMs in *Eng^2fl/2fl^*;R26CreER were induced by VEGF, the lesions were thus always located at the AAV-VEGF injection sites. Further, the AVM phenotype was also detected around the ear-tag wound of all TM treated *Eng^2fl/2fl^*;*R26*CreER mice (Figure S4A). No AVM phenotype was found in the contralateral uninjured ear (data not shown) and the intestine (Figure S4B). Taken together, global conditional *Eng* deletion in adult mice resulted in de novo AVMs in the brain angiogenic foci and around the skin wound.

### No AVM found in *Eng^2fl/2fl^;LysM*Cre mice

**Figure 3:**
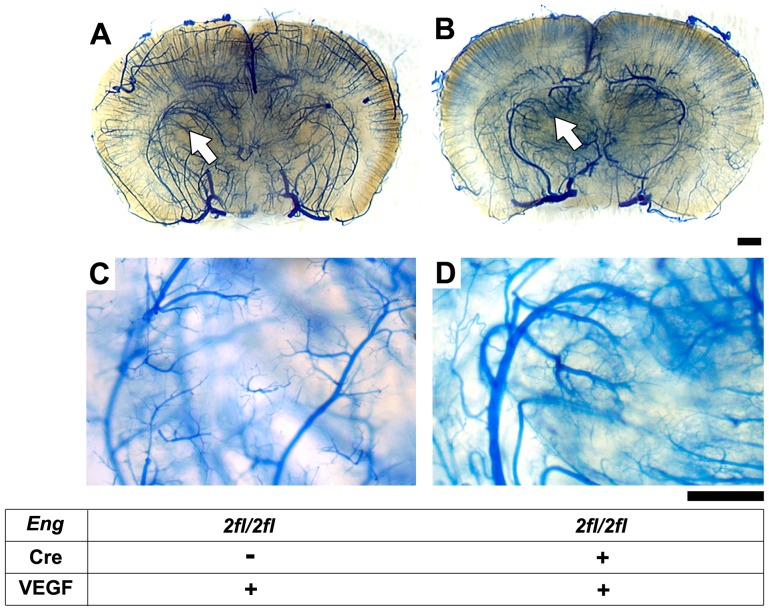
AVM phenotype was not detected in the brain angiogenic region of *Eng^2fl/2fl^;LysM-*Cre mice. Representative images of the latex-casted cerebrovasculature in (A) *Eng^2fl/2fl^* and (B) *Eng^2fl/2fl^*;*LysM*Cre adult mice 8 weeks after focal VEGF stimulation. C and D: High magnification view of the angiogenic foci (arrows) shown in A and B. Latex was present in arteries only. No vein and enlarged and tangled vessels were detected. Scale bars: 1 mm in A and B and 500 µm in C and D.

We have previously shown that bone marrow-derived macrophages home to the brain angiogenic foci [18]. *Eng^+/−^* mice and WT mice transplanted with *Eng^+/−^* bone marrow developed a similar degree of dysplasia after VEGF stimulation [17]. These data suggest that bone marrow-derived macrophages may play a role in brain AVM development. To test if *Eng* deletion in macrophages is sufficient to induce brain AVMs, we utilized the *LysM*-Cre mouse [15] that specifically mediates target gene recombination in lysozyme M-positive macrophages. *Eng^2fl/2fl^;LysM-*Cre mice are born with the expected Mendelian ratio and develop normally into the adult stage. Injection of AAV-VEGF into the basal ganglia of 8 to 10-week-old *Eng^2fl/2fl^;LysM-*Cre mice did not induce AVM phenotype in the brain (Figure 3). Similarly, no AVM phenotype was observed around the ear wound (Figure S4C) and intestine (Figure S4D). Real-time genomic DNA qPCR analysis confirmed that more than 90% *Eng* was deleted in macrophages of *Eng^2fl/2fl^;LysM-*Cre mice (Figure S5), indicating that the lack of AVM phenotype was not due to inefficient gene deletion. Thus, these data suggest that *Eng* deletion in lysozyme M-positive macrophages alone was not sufficient to initiate AVM formation.

### *Eng*-null endothelial cells detected in brain AVM vessels

**Figure 4:**
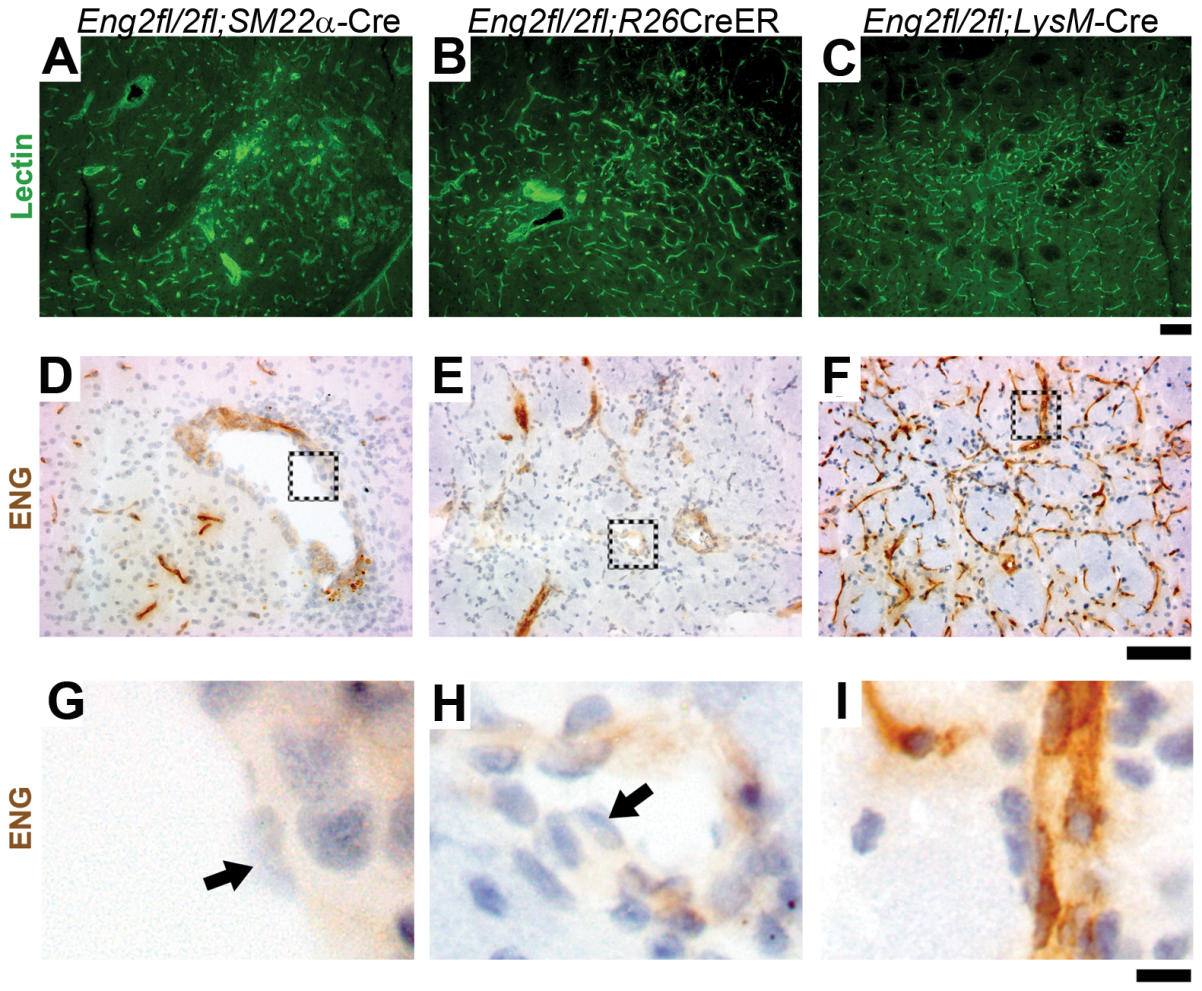
ENG-null endothelial cells in dysplastic vessels. Representative images of lectin-stained brain sections from (A) AVM lesion of 5-week-old *Eng^2fl/2fl^*;*SM22α*-Cre, (B) VEGF-induced angiogenic focus of TM-treated adult *Eng^2fl/2fl^*;*R26*CreER, and (C) VEGF-stimulated angiogenic focus of adult *Eng^2fl/2fl^*;*LysM-*Cre mice. ENG expression in (D) *Eng^2fl/2fl^*;*SM22α-*Cre, (E) *Eng^2fl/2fl^*;*R26*CreER, and (F) *Eng^2fl/2fl^*;*LysM-*Cre brain. G-I: Enlarged images of the dotted boxes shown in D-F. Arrows indicate ENG-negative endothelial cells. Scale bars: 100 µm in A–F and 10 µm in G–I.

**Figure 5:**
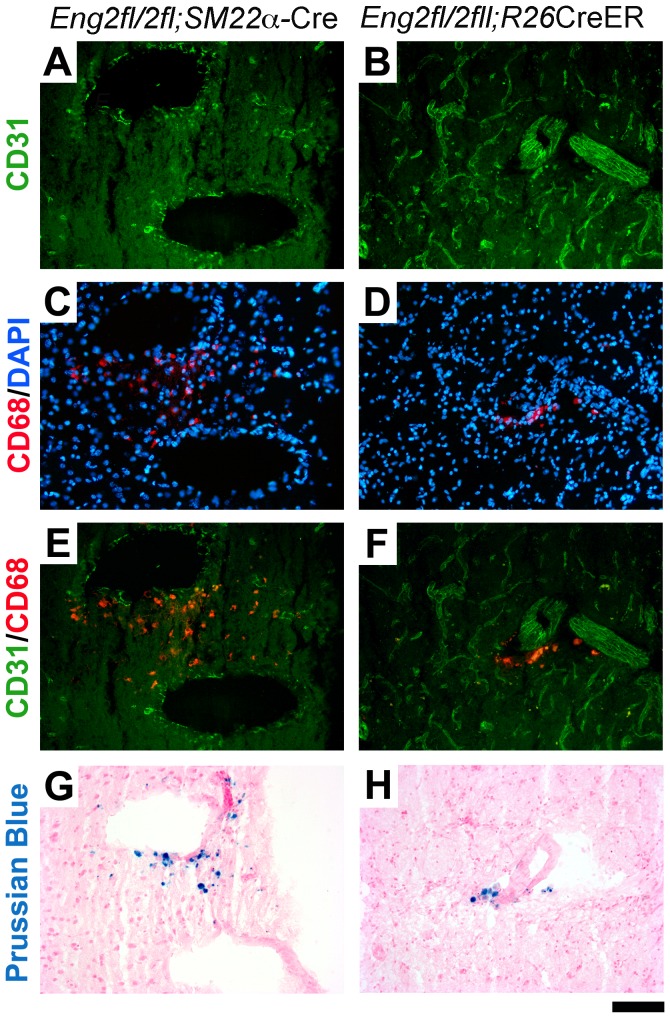
Macrophage infiltration and microhemorrhage in brain AVM lesions of *Eng^2fl/2fl^*;*SM22α*Cre and *Eng^2fl/2fl^*;*R26*CreER mice. Dysplastic vessels were present in the brain of (A) *Eng^2fl/2fl^*;*SM22α*-Cre and (B) *Eng^2fl/2fl^*;*R26*CreER mice. Endothelial cells were visualized by immunostaining using an antibody specific to CD31 (an endothelial cell-specific marker). C and D: Macrophages were detected by immunostaining using a CD68-specific antibody. The nuclei were stained by DAPI. E and F: Macrophages localized around large dysplastic vessels. G and H: Iron deposits, an indicator of hemorrhage, identified by Prussian blue. Scale bar: 100 µm.

Dilated and dysmorphic (dysplasia) vessels were found in lectin-stained brain sections of 5-week-old *Eng^2fl/2fl^;SM22α*-Cre mice (Figure 4A) and around the brain angiogenic region of TM-treated adult *Eng^2fl/2fl^;R26*CreER mice (Figure 4B), but not in the brain angiogenic region of adult *Eng^2fl/2fl^;LysM-*Cre mice (Figure 4C). Endothelial ENG expression was greatly reduced in the lesions of brain sections from *Eng^2fl/2fl^;SM22α*-Cre and *Eng^2fl/2fl^;R26*CreER mice (Figure 4D and 4E). Endothelial ENG expression in the angiogenic foci of *Eng^2fl/2fl^;LysM-*Cre mice did not change (Figure 4F and 4I). Notably, ENG-null endothelial cells were found in large dysplastic vessels in cerebral lesions in both *Eng^2fl/2fl^;SM22α*-Cre (Figure 4G) and *Eng^2fl/2fl^;R26*CreER (Fig. 5H) mice. Further, similar to the phenotype of the *Alk1*-deficient model (HHT2 model) [19] and human unruptured brain AVMs [20], [21], macrophages and microhemorrhage were observed around dysplastic vessels in the brain of *Eng^2fl/2fl^;SM22α*-Cre and *Eng^2fl/2fl^;R26*CreER mice (Figure 5). Thus, all of the evidence indicates that homozygous deletion of *Eng* in endothelial cells is necessary for the development of macroscopic AVM-like vessels with arteriovenous (A–V) shunts in the mouse brain.

## Discussion

In this study, we established two novel HHT1 brain AVM mouse models: one, developmental onset, and the other, adult onset. These models are better suited for mechanistic studies and new drug tests than the existing HHT brain AVM models we and others have developed [9]–[12]. We have also shown that endothelial homozygous *Eng* deletion is required for the development of the HHT1 brain AVM phenotype, whereas deletion of *Eng* in macrophages is insufficient.

As a model for investigating AVM pathogenesis and testing novel therapies, our HHT1 developmental model created in *Eng^2fl/2fl^;SM22α*-Cre mice has some advantages compared to the two HHT2 developmental brain AVM models developed in *Alk1^2fl/2fl^*;L1Cre mice [9] and *Alk1^2fl/2fl^;SM22α*-Cre mice [10]. The *Alk1^2fl/2fl^*;L1Cre mice died at postnatal day 5 due to massive intracranial hemorrhage [9]. More than 50% of *Alk1^2fl/2fl^;SM22α*-Cre mice died before 2 weeks of age [10]. Such a short lifespan limits their use in testing new therapies. In contrast, we did not observe any lethality in our *Eng^2fl/2fl^*;*SM22α*-Cre model until postnatal week 3. About 30% of the mice died between 3 and 6 weeks due to cerebral and/or gastrointestinal hemorrhage. The penetrance of the brain AVM phenotype was about 90% at 5 weeks of age. Further, there is no other manipulation, such as angiogenic stimulation, needed to initiate brain AVM development in addition to gene deletion. Thus, brain AVM initiation and progression in *Eng^2fl/2fl^*;*SM22α*-Cre mice more closely resemble spontaneously developed human brain AVM, making this model more suitable for studying brain AVM pathogenesis and testing new therapies. Since the lesion size, location, and number in this model are highly variable, identifying the location of the AVM lesion and quantifying the dysplasia index by tissue section would be time consuming and challenging. However, the AVM lesions were only detected in *Eng^2fl/2fl^*;*SM22α-*Cre mice, and not in *Eng^2fl/2fl^* or *Eng^+/2fl^*;*SM22α-*Cre mice (Figure 1). We recorded the presence of large AVMs in a whole-mount brain preparation following latex casting in 90% of *Eng^2fl/2fl^*;*SM22α-*Cre mice. This method detected lesions with A–V shunts (latex dye presented in the veins). More than 80% of the mice had one lesion, and the remaining 20%, 2–3 lesions. Thus, comparing the number of mice with lesions in the treated and control groups could be used to evaluate the therapeutic efficacy of new test drugs. In addition, since *Eng^2fl/2fl^*;*SM22α*-Cre mice died at various times (Figure 1G), analysis of the mortality rate could be another means of evaluating a test drug's effectiveness. More detailed quantification would be extremely time consuming due to the non-specific location of the lesions, which is a limitation of the *Eng^2fl/2fl^*;*SM22α-*Cre model.

The capillary level dysplasia in the brain of *Eng^+/−^* mice described previously does not closely resemble the human brain AVM phenotype [12]. The major drawback of our published adult onset brain AVM model is that a Cre-expressing adenoviral vector was used to induce focal *Alk1* deletion [11]. Because the adenoviral vector induces potent local inflammation [22], it limits the use of this model to the study of the role of inflammation in brain AVM phenotype development and progression. We have overcome this limitation in the present model by using the *R26*CreER transgene to achieve conditional *Eng* deletion. Unlike *Alk1^2fl/2fl^*;*R26*CreER mice that exhibited 100% lethality 2 to 3 weeks after TM-induced gene deletion [9], *Eng^2fl/2fl^*;*R26*CreER mice treated with our selected TM dose survived more than 2 months after the induction of *Eng* deletion. After intra-brain injection of AAV-VEGF, *Eng^2fl/2fl^*;*R26*CreER mice developed the phenotype resembling human brain AVM. AAV causes fewer host inflammatory and immune responses than adenoviral vectors [22]. Therefore, this is a better model for studying the role of inflammation in brain AVM pathogenesis than the model induced by Ad-Cre/AAV-VEGF-mediated focal *Alk1*-deletion and angiogenesis [11], [19].

We detected AVM only in the intestines of *Eng^2fl/2fl^*;*SM22α*-Cre mice, but not in *Eng^2fl/2fl^*;*R26*CreER mice. Interestingly, adult *Alk1^2fl/2fl^*;*R26*CreER mice developed AVMs in their lungs and intestines after TM treatment [9]. Additionally, *Eng^2fl/2fl^*;*R26*CreER mice survived 2 months after TM treatment, whereas all *Alk1^2fl/2fl^*;*R26*CreER mice died within 2 to 3 weeks after TM. We hypothesize that ineffective gene deletion in *Eng^2fl/2fl^*;*R26*CreER was responsible for some of the phenotypic difference between *Eng^2fl/2fl^*;*R26*CreER and *Alk1^2fl/2fl^*;*R26*CreER. We found in our previous study that stereotactic injection of the same dose of Ad-Cre resulted in 16% *Alk1* deletion in the brain of *Alk1^2fl/2fl^* mice and less than 1% *Eng* deletion in *Eng^2fl/2fl^* mice [13]. In a future study, we will use a more powerful gene deletion system to test this hypothesis.

The prevailing view regarding AVM manifestation in HHT is that it is caused by haploinsufficiency of one of its causative genes. However, *ENG* haploinsufficiency in endothelial cells in human HHT lesions appears to be insufficient for AVM development [23]. We found that *Eng* homozygous deletion in endothelial cells seems to be required for brain AVM formation. Both *SM22α*-Cre and *R26*CreER mediate recombination of loxP sites in endothelial cells [9], [10]. The fact that the brain AVM phenotype developed only in *Eng^2fl/2fl^*;*SM22α*-Cre and *Eng^2fl/2fl^*;*R26*CreER mice, but not in *Eng^+/2fl^*;*SM22α*-Cre and *Eng^+/2fl^*;*R26*CreER mice, suggests that *Eng* homozygous deletion is necessary. These data are consistent with our previous findings that: (1) only capillary level (microscopic level) vascular dysplasia developed in the brain of adult *Eng^+/−^* and *Alk1^+/−^* mice after intra-brain injection of AAV-VEGF [12]; and (2) *Eng* homozygous deletion of ∼1% endothelial cells in the adult mouse brain was sufficient to induce the macroscopic level of cerebrovascular dysplasia phenotype [13]. Hence, loss of function of the normal allele in the endothelium of HHT patients might be necessary for AVM formation. Many factors contributing to the inactivation or downregulation of the normal allele's function have been suggested, such as focal second-hit somatic mutations, shedding of ENG from endothelial cells during inflammation [24], [25], and reduced endothelial ENG signaling due to increased levels of soluble endoglin (sENG) [26].

In addition, recent studies indicate that bone marrow-derived cells play a crucial role in tissue repair and angiogenesis [18], [27], [28]. We have shown that *Eng^+/−^* mice and WT mice transplanted with *Eng^+/−^* bone marrow developed a similar degree of capillary level cerebrovascular dysplasia after VEGF stimulation [17]. These data suggest that bone marrow cells are involved in brain AVM pathogenesis. Macrophage is the major bone marrow-derived cell type detected in the brain angiogenic foci [17], [18], [28]. To explore the role of macrophages in brain AVM development, we used *LysM*-Cre transgenic mouse lines to delete *Eng* in macrophages. *LysM*-Cre selectively induces gene recombination in lysozyme M-positive macrophages [15]. Mice with *LysM*-Cre-driven *Eng* deletion during the embryonic developmental stage did not develop the brain AVM phenotype even after VEGF stimulation. Therefore, deletion of *Eng* in microphages alone is not sufficient for brain AVM formation.

However, this finding does not rule out a potential contributory role of macrophage endoglin during AVM pathogenesis. *Eng* deficiency appears to impair monocyte adhesion and migration. The homing ability of ENG-deficient HHT1 monocytes to the infarcted murine heart is impaired [27], [29]. *Eng* deficiency in endothelial cells reduces leukocyte adhesion and transmigration [30] and impairs the endothelial-autonomous capacity to up-regulate SDF1 expression in response to hindlimb ischemic injury [31]. However, macrophage load is higher in unruptured human brain AVM lesions than in control vessels [20], [21] and there is also high monocyte/macrophage infiltration in HHT skin lesions [32]. We previously found that a systemic reduction in endoglin levels led to a temporal difference in macrophage responses. *Eng^+/−^* mice had fewer CD68^+^ cells in the peri-infarct area at 3 days but more CD68^+^ cells at 60 days after permanent occlusion of a distal middle cerebral artery [33]. It appears that in the early stages of injury, there are fewer monocytes homing to the injury site. At the late stage when recovery normally occurs, more macrophages persist around abnormal vessels, potentially promoting disease progression. In addition, extravasation of blood content from dysplastic AVM vessels could also attract macrophage infiltration [19], [21]. Indeed, macrophages were mostly co-localized with iron deposits around the dysplastic vessels in the two models we present in this manuscript (Figure 5). Further study will be needed to understand how macrophages enter the AVM lesion, and the correlation between macrophage load and patient outcomes.

A limitation of this study is that vascular leakage and macrophage infiltration were not quantified in these models. However, we did observe that these phenotypes were not as severe as in the *Alk1*-deficient model we previously reported [19]. The potential reasons include: (1) the vascular defect takes longer to develop in Eng-deficient vessels than in Alk1-deficient vessels; and (2) the gene deletion in our previous Alk1 model was induced by Ad-Cre, and as a result, the inflammatory response to adenoviral vector could have resulted in increased macrophage infiltration and more severe vascular leakage [19]. In addition, ineffective gene deletion in *Eng^2fl/2fl^*;*R26*CreER could also be a reason for a milder phenotype. Stereotactic injection of the same dose of Ad-Cre resulted in 16% *Alk1* deletion in the brain of *Alk1^2fl/2fl^* mice and less than 1% *Eng* deletion in *Eng^2fl/2fl^* mice [13]. In the future, we will observe the lesion for a longer period of time, or use a more powerful cre system.

Although many advances have been made in the HHT field, the underlying molecular and cellular mechanisms for AVM formation are still unclear. Our novel mouse brain AVM models would be valuable resources for dissecting brain AVM pathogenesis. Studying more common familial brain AVM cases will help us understand unknown causes of sporadic brain AVMs. The detailed long-term natural history of these models would also allow us to understand the progression of brain AVM, paving the way towards improving patient care. Further, preclinical studies using these animals will lead to the design of a safer and more effective pharmacological therapy for HHT brain AVM patients, which may also apply to patients with sporadic brain AVM.

## Supporting Information

Table S1 **Primers used for real-time quantitative genomic DNA PCR analysis.** Matrix metalloproteinase 9 (*Mmp9*) was used as an internal quantitative control.
(DOCX)

Figure S1 **Conditional** ***Eng*** **deletion in the adult mouse using the** ***R26*** **CreER transgenic mouse.** A: Experimental design. TM was injected to *Eng^2fl/2fl^*;*R26*Cre-ER mice i.p. once per day for 3 consecutive days. AAV-VEGF was injected into the right basal ganglia at the time when the first dose of TM was given. Samples were collected for phenotype analysis 8 weeks after the TM and AAV-VEGF injection. B: Quantification of WT *Eng* (2fl) allele in the genomic DNA isolated from the brain 8 weeks after the first tamoxifen (TM) injection. n = 3 per group.
(TIF)

Figure S2 ***SM22α*** **-Cre-driven** ***Eng*** **deletion resulted in AVMs in the postnatal spinal cord and intestine.** AVM phenotypes in the (C) spinal cord and (F) intestine of *Eng^2fl/2fl^*;*SM22α*-Cre mice, but not in those of control mice (A, B, D, and E). Arteries (a) and veins (v) are shown in dark and light blue, respectively, in the intestine of *Eng^2fl/2fl^*;*SM22α*-Cre mice. Scale bars: 1 mm.
(TIF)

Figure S3 **AVMs and microhemorrhages found in the brain and spinal cord of 5-week-old** ***Eng^2fl/2fl^*** **;** ***SM22α*** **-Cre mice.** Examples of (A) superficially and (B) deeply located brain AVMs. Hemorrhages (arrows) detected in some lesions of the (C) brain and (D) spinal cord. a: Artery. v: Vein. Scale bars: 1 mm.
(TIF)

Figure S4 ***R26*** **CreER-mediated conditional** ***Eng*** **deletion induced de novo skin AVMs around the ear wound.** A: AVM vessels around the ear wound (*) of tamoxifen-treated *Eng^2fl/2fl^*;*R26*CreER mice. B: No arteriovenous (A–V) shunts in the intestine of *Eng^2fl/2fl^*;*R26*CreER mice 8 weeks after tamoxifen treatment. No abnormal vascular phenotype observed around the (C) ear wound (*) and in the (D) intestine of *Eng^2fl/2fl^*;*LysM-*Cre mice. Scale bars: 1 mm.
(TIF)

Figure S5 ***LysM*** **-Cre induced effective** ***Eng*** **deletion in macrophages.** Relative amount of the targeted *Eng* conditional 2fl allele in *Eng^2fl/2fl^*;*LysM-*Cre macrophages compared to that of *Eng^2fl/2fl^* macrophages. n = 3 per group.
(TIF)

## References

[1] McAllister KA, Grogg KM, Johnson DW, Gallione CJ, Baldwin MA, et al (1994) Endoglin, a TGF-beta binding protein of endothelial cells, is the gene for hereditary haemorrhagic telangiectasia type 1. Nat Genet 8: 345–351. 7894484 10.1038/ng1294-345

[2] Shovlin CL, Guttmacher AE, Buscarini E, Faughnan ME, Hyland RH, et al (2000) Diagnostic criteria for hereditary hemorrhagic telangiectasia (Rendu-Osler-Weber syndrome). Am J Med Genet 91: 66–67. 10751092 10.1002/(sici)1096-8628(20000306)91:1<66::aid-ajmg12>3.0.co;2-p

[3] Johnson DW, Berg JN, Baldwin MA, Gallione CJ, Marondel I, et al (1996) Mutations in the activin receptor-like kinase 1 gene in hereditary haemorrhagic telangiectasia type 2. Nat Genet 13: 189–195. 8640225 10.1038/ng0696-189

[4] Kim H, Marchuk DA, Pawlikowska L, Chen Y, Su H, et al.. (2008) Genetic considerations relevant to intracranial hemorrhage and brain arteriovenous malformations. Acta Neurochir Suppl 105: 199–206. 10.1007/978-3-211-09469-3_38 PMC2640934 19066109

[5] Kim H, Su H, Weinsheimer S, Pawlikowska L, Young WL (2011) Brain arteriovenous malformation pathogenesis: a response-to-injury paradigm. Acta Neurochir Suppl 111: 83–92. 10.1007/978-3-7091-0693-8_14 PMC3187860 21725736

[6] Easey AJ, Wallace GM, Hughes JM, Jackson JE, Taylor WJ, et al (2003) Should asymptomatic patients with hereditary haemorrhagic telangiectasia (HHT) be screened for cerebral vascular malformations? Data from 22,061 years of HHT patient life. J Neurol Neurosurg Psychiatry 74: 743–748. 12754343 10.1136/jnnp.74.6.743 PMC1738468

[7] Han PP, Ponce FA, Spetzler RF (2003) Intention-to-treat analysis of Spetzler-Martin grades IV and V arteriovenous malformations: natural history and treatment paradigm. J Neurosurg 98: 3–7. 12546345 10.3171/jns.2003.98.1.0003

[8] Bambakidis NC, Cockroft K, Connolly ES, Amin-Hanjani S, Morcos J, et al (2013) Preliminary results of the ARUBA sudy. Neurosurgery 73: E379–381. 23867264 10.1227/NEU.0000000000000067

[9] Park SO, Wankhede M, Lee YJ, Choi EJ, Fliess N, et al (2009) Real-time imaging of de novo arteriovenous malformation in a mouse model of hereditary hemorrhagic telangiectasia. J Clin Invest 119: 3487–3496. 19805914 10.1172/JCI39482 PMC2769195

[10] Milton I, Ouyang D, Allen CJ, Yanasak NE, Gossage JR, et al (2012) Age-dependent lethality in novel transgenic mouse models of central nervous system arteriovenous malformations. Stroke 43: 1432–1435. 22328553 10.1161/STROKEAHA.111.647024

[11] Walker EJ, Su H, Shen F, Choi EJ, Oh SP, et al (2011) Arteriovenous malformation in the adult mouse brain resembling the human disease. Ann Neurol 69: 954–962. 21437931 10.1002/ana.22348 PMC3117949

[12] Hao Q, Zhu Y, Su H, Shen F, Yang GY, et al (2010) VEGF induces more severe cerebrovascular dysplasia in Endoglin^+/−^ than in Alk1^+/−^ mice. Transl Stroke Res 1: 197–201. 20640035 10.1007/s12975-010-0020-x PMC2902730

[13] Choi EJ, Walker EJ, Shen F, Oh SP, Arthur HM, et al (2012) Minimal homozygous endothelial deletion of Eng with VEGF stimulation is sufficient to cause cerebrovascular dysplasia in the adult mouse. Cerebrovasc Dis 33: 540–547. 22571958 10.1159/000337762 PMC3569027

[14] Allinson KR, Carvalho RL, van den Brink S, Mummery CL, Arthur HM (2007) Generation of a floxed allele of the mouse Endoglin gene. Genesis 45: 391–395. 17506087 10.1002/dvg.20284 PMC2077828

[15] Clausen BE, Burkhardt C, Reith W, Renkawitz R, Forster I (1999) Conditional gene targeting in macrophages and granulocytes using LysMcre mice. Transgenic Res 8: 265–277. 10621974 10.1023/a:1008942828960

[16] Su H, Lu R, Kan YW (2000) Adeno-associated viral vector-mediated vascular endothelial growth factor gene transfer induces neovascular formation in ischemic heart. Proc Natl Acad Sci U S A 97: 13801–13806. 11095751 10.1073/pnas.250488097 PMC17656

[17] Choi EJ, Walker EJ, Degos V, Jun K, Kuo R, et al (2013) Endoglin deficiency in bone marrow is sufficient to cause cerebrovascular dysplasia in the adult mouse after vascular endothelial growth factor stimulation. Stroke 44: 795–798. 23306322 10.1161/STROKEAHA.112.671974 PMC3582755

[18] Hao Q, Liu J, Pappu R, Su H, Rola R, et al (2008) Contribution of bone marrow-derived cells associated with brain angiogenesis is primarily through leucocytes and macrophages. Arterioscler Thromb Vasc Biol 28: 2151–2157. 18802012 10.1161/ATVBAHA.108.176297 PMC2610019

[19] Chen W, Guo Y, Walker EJ, Shen F, Jun K, et al (2013) Reduced mural cell coverage and impaired vessel integrity after angiogenic stimulation in the *Alk1*-deficient brain. Arterioscler Thromb Vasc Biol 33: 305–310. 23241407 10.1161/ATVBAHA.112.300485 PMC3569037

[20] Chen Y, Zhu W, Bollen AW, Lawton MT, Barbaro NM, et al (2008) Evidence of inflammatory cell involvement in brain arteriovenous malformations. Neurosurgery 62: 1340–1349. 18825001 10.1227/01.neu.0000333306.64683.b5 PMC2582017

[21] Guo Y, Saunders T, Su H, Kim H, Akkoc D, et al (2012) Silent intralesional microhemorrhage as a risk factor for brain arteriovenous malformation rupture. Stroke 43: 1240–1246. 22308253 10.1161/STROKEAHA.111.647263 PMC3335931

[22] Zaiss AK, Liu Q, Bowen GP, Wong NC, Bartlett JS, et al (2002) Differential activation of innate immune responses by adenovirus and adeno-associated virus vectors. J Virol 76: 4580–4590. 11932423 10.1128/JVI.76.9.4580-4590.2002 PMC155101

[23] Bourdeau A, Cymerman U, Paquet ME, Meschino W, McKinnon WC, et al (2000) Endoglin expression is reduced in normal vessels but still detectable in arteriovenous malformations of patients with hereditary hemorrhagic telangiectasia type 1. Am J Pathol 156: 911–923. 10702408 10.1016/S0002-9440(10)64960-7 PMC1876827

[24] Li C, Guo B, Ding S, Rius C, Langa C, et al (2003) TNF alpha down-regulates CD105 expression in vascular endothelial cells: a comparative study with TGF beta 1. Anticancer Res 23: 1189–1196. 12820370

[25] Mahmoud M, Allinson KR, Zhai Z, Oakenfull R, Ghandi P, et al (2010) Pathogenesis of arteriovenous malformations in the absence of endoglin. Circ Res 106: 1425–1433. 20224041 10.1161/CIRCRESAHA.109.211037

[26] Chen Y, Hao Q, Kim H, Su H, Letarte M, et al (2009) Soluble endoglin modulates aberrant cerebral vascular remodeling. Ann Neurol 66: 19–27. 19670444 10.1002/ana.21710 PMC2773229

[27] van Laake LW, van den Driesche S, Post S, Feijen A, Jansen MA, et al (2006) Endoglin has a crucial role in blood cell-mediated vascular repair. Circulation 114: 2288–2297. 17088457 10.1161/CIRCULATIONAHA.106.639161

[28] Hao Q, Su H, Palmer D, Sun B, Gao P, et al (2011) Bone marrow-derived cells contribute to vascular endothelial growth factor-induced angiogenesis in the adult mouse brain by supplying matrix metalloproteinase-9. Stroke 42: 453–458. 21164138 10.1161/STROKEAHA.110.596452 PMC3026909

[29] Post S, Smits AM, van den Broek AJ, Sluijter JP, Hoefer IE, et al (2010) Impaired recruitment of HHT-1 mononuclear cells to the ischaemic heart is due to an altered CXCR4/CD26 balance. Cardiovasc Res 85: 494–502. 19762327 10.1093/cvr/cvp313

[30] Rossi E, Sanz-Rodriguez F, Eleno N, Duwell A, Blanco FJ, et al (2013) Endothelial endoglin is involved in inflammation: role in leukocyte adhesion and transmigration. Blood 121: 403–415. 23074273 10.1182/blood-2012-06-435347

[31] Young K, Conley B, Romero D, Tweedie E, O'Neill C, et al (2012) BMP9 regulates endoglin-dependent chemokine responses in endothelial cells. Blood 120: 4263–4273. 23018639 10.1182/blood-2012-07-440784 PMC3501721

[32] Braverman IM, Keh A, Jacobson BS (1990) Ultrastructure and three-dimensional organization of the telangiectases of hereditary hemorrhagic telangiectasia. J Invest Dermatol 95: 422–427. 2212727 10.1111/1523-1747.ep12555569

[33] Shen F, Degos V, Han Z, Choi EJ, Young WL, et al (2013) Endoglin deficiency exacerbates ischemis brain injury (Abstract). Stroke 44: ATMP69.

