# Supplementary material for: Novel Brain Arteriovenous Malformation Mouse Models for Type 1 Hereditary Hemorrhagic Telangiectasia
Source: PLoS One. 2014 Feb 10;9(2):e88511. doi: 10.1371/journal.pone.0088511 (PMC3919779; doi:10.1371/journal.pone.0088511)
Supplement: Table S1 — Primers used for real-time quantitative genomic DNA PCR analysis. Matrix metalloproteinase 9 (Mmp9) was used as an internal quantitative control. (DOCX) [file pone.0088511.s001.docx]

**Table S1. Primers used for real-time quantitative genomic DNA PCR analysis.**

| **Gene** | **Forward Primer** | **Reverse Primer** |
| --- | --- | --- |
| *Eng* | GACGCCATTCTCATCCTGC | CCACGCCTTTGTCCTTGC |
| *Mmp9* | GTGGGACCATCATAACATCACA | CTCGCGGCAAGTCTTCAGAGTA |

Matrix metalloproteinase 9 (*Mmp9*) was used as an internal quantitative control.
