# Supplementary figures and images for: Novel Brain Arteriovenous Malformation Mouse Models for Type 1 Hereditary Hemorrhagic Telangiectasia
Source: PLoS One. 2014 Feb 10;9(2):e88511. doi: 10.1371/journal.pone.0088511 (PMC3919779; doi:10.1371/journal.pone.0088511)

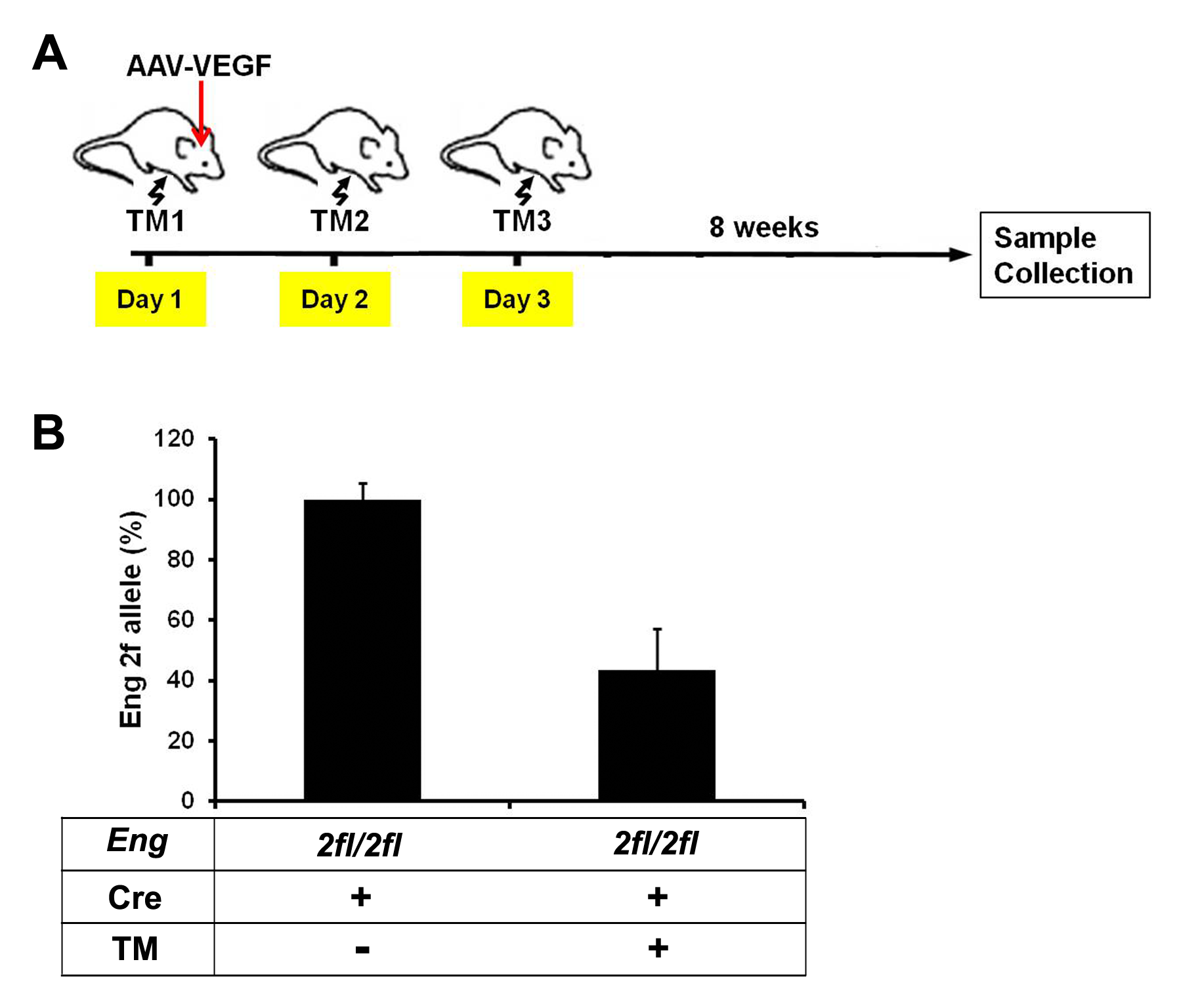

Supplement: Figure S1 — Conditional Eng deletion in the adult mouse using the R26 CreER transgenic mouse. A: Experimental design. TM was injected to Eng2fl/2fl;R26Cre-ER mice i.p. once per day for 3 consecutive days. AAV-VEGF was injected into the right basal ganglia at the time when the first dose of TM was given. Samples were collected for phenotype analysis 8 weeks after the TM and AAV-VEGF injection. B: Quantification of WT Eng (2fl) allele in the genomic DNA isolated from the brain 8 weeks after the first tamoxifen (TM) injection. n = 3 per group. (TIF) [file pone.0088511.s002.tif]

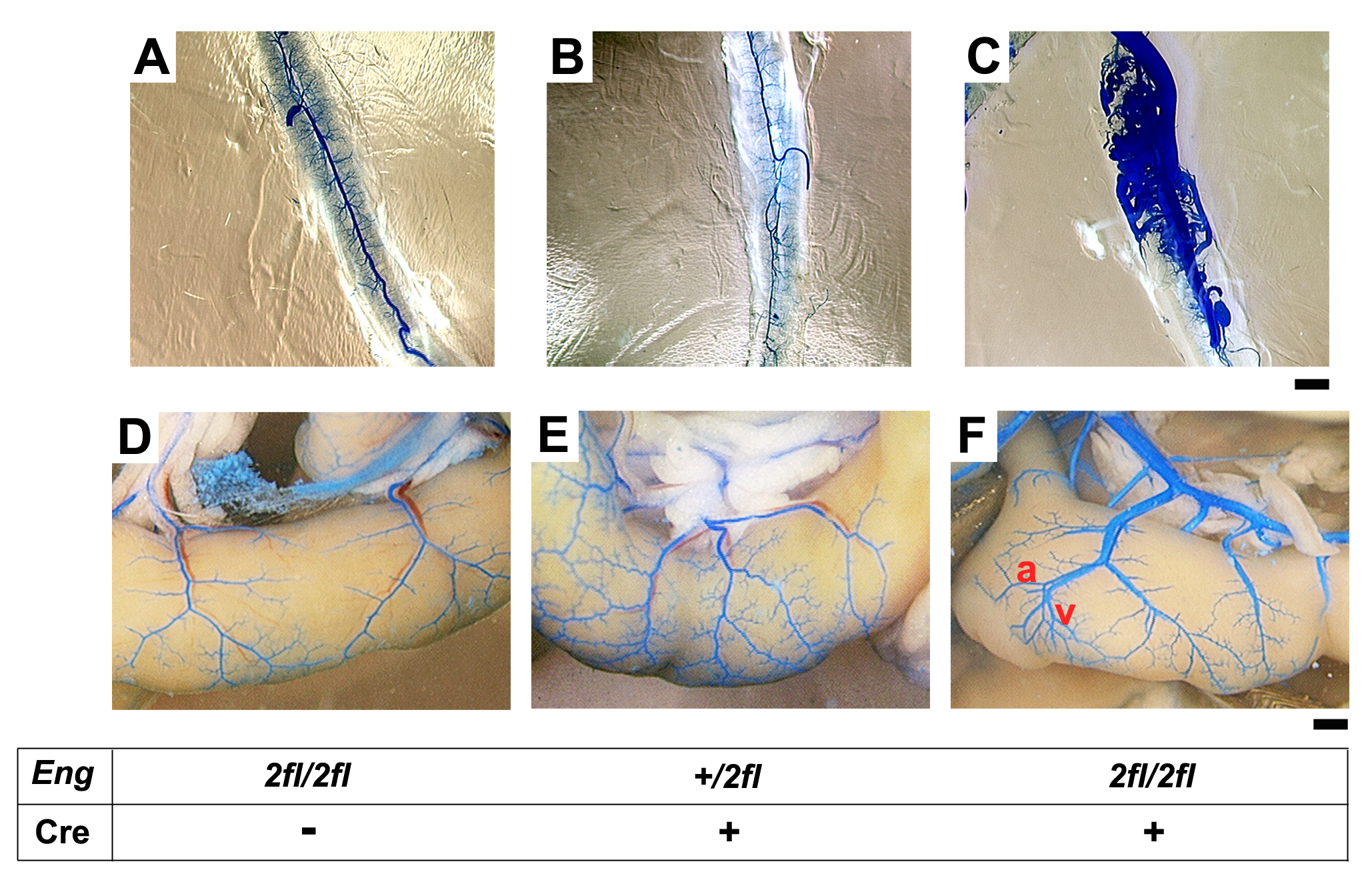

Supplement: Figure S2 — SM22α -Cre-driven Eng deletion resulted in AVMs in the postnatal spinal cord and intestine. AVM phenotypes in the (C) spinal cord and (F) intestine of Eng2fl/2fl;SM22α-Cre mice, but not in those of control mice (A, B, D, and E). Arteries (a) and veins (v) are shown in dark and light blue, respectively, in the intestine of Eng2fl/2fl;SM22α-Cre mice. Scale bars: 1 mm. (TIF) [file pone.0088511.s003.tif]

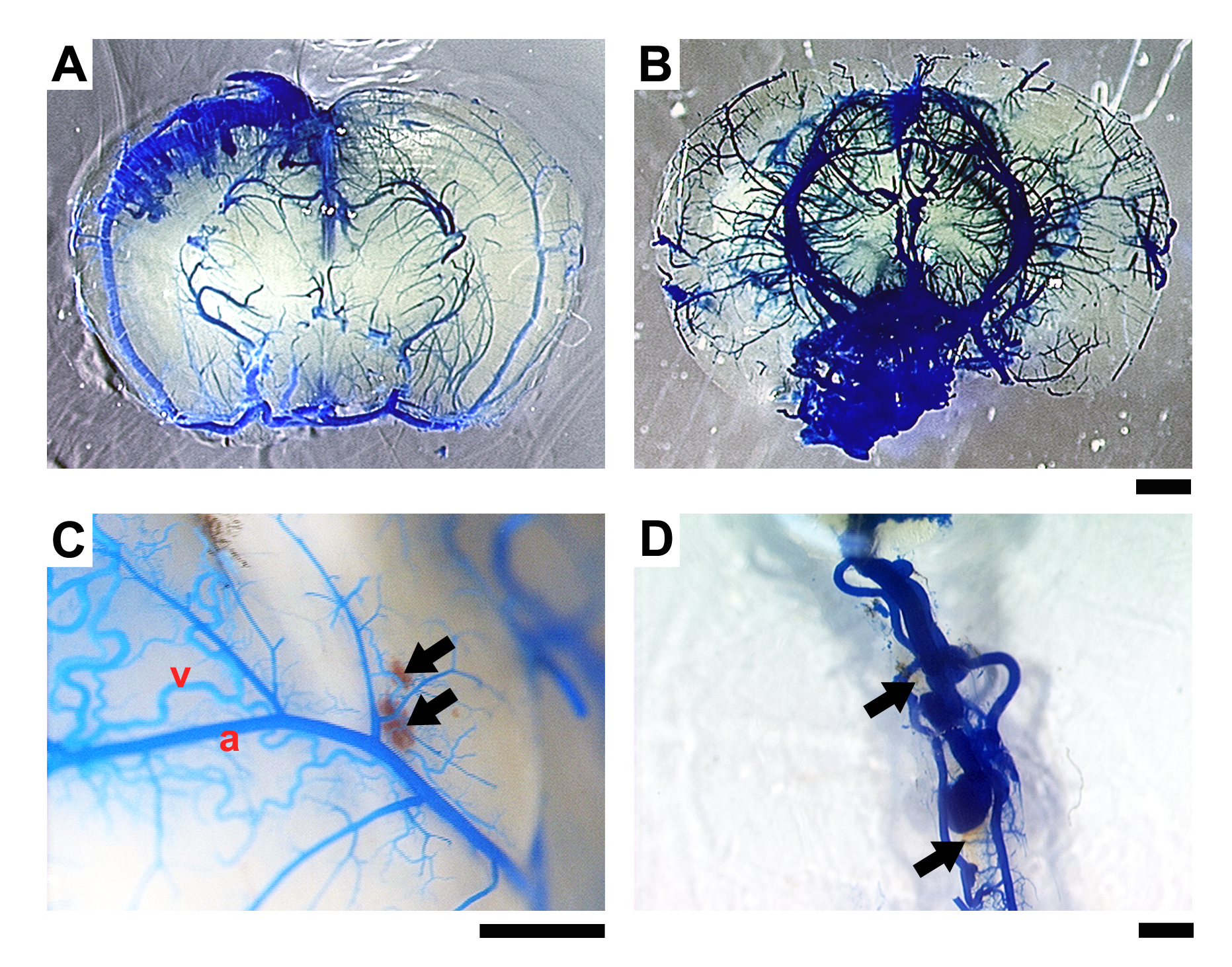

Supplement: Figure S3 — AVMs and microhemorrhages found in the brain and spinal cord of 5-week-old Eng2fl/2fl ; SM22α -Cre mice. Examples of (A) superficially and (B) deeply located brain AVMs. Hemorrhages (arrows) detected in some lesions of the (C) brain and (D) spinal cord. a: Artery. v: Vein. Scale bars: 1 mm. (TIF) [file pone.0088511.s004.tif]

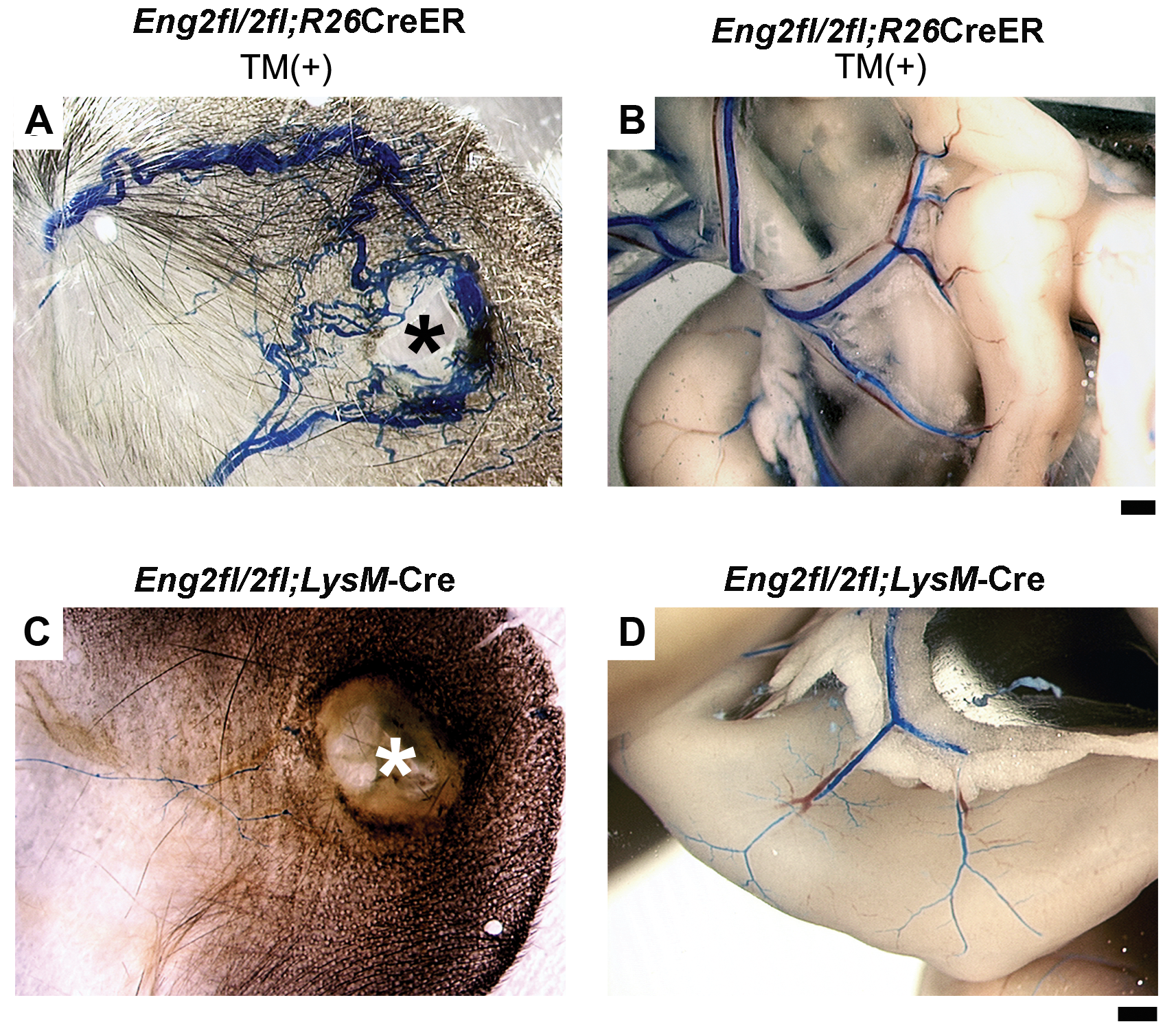

Supplement: Figure S4 — R26 CreER-mediated conditional Eng deletion induced de novo skin AVMs around the ear wound. A: AVM vessels around the ear wound (*) of tamoxifen-treated Eng2fl/2fl;R26CreER mice. B: No arteriovenous (A–V) shunts in the intestine of Eng2fl/2fl;R26CreER mice 8 weeks after tamoxifen treatment. No abnormal vascular phenotype observed around the (C) ear wound (*) and in the (D) intestine of Eng2fl/2fl;LysM-Cre mice. Scale bars: 1 mm. (TIF) [file pone.0088511.s005.tif]

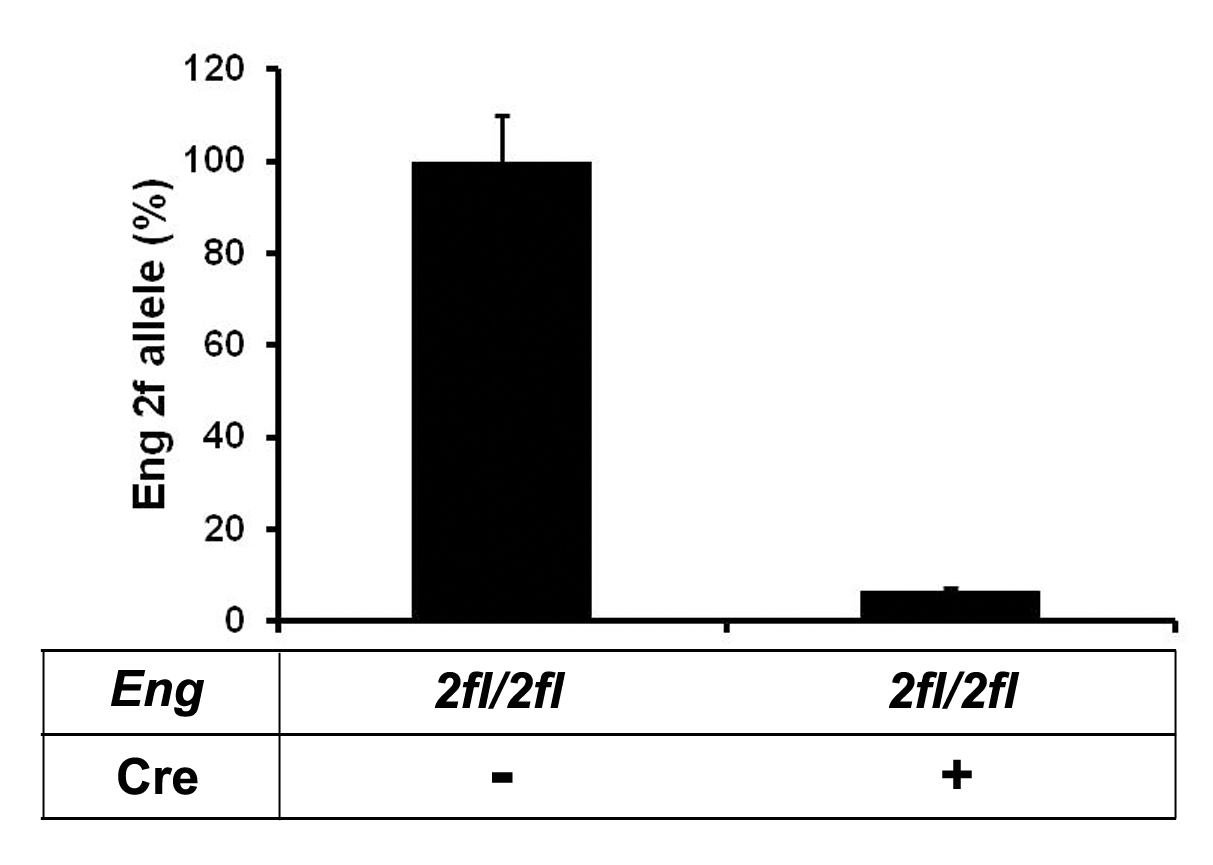

Supplement: Figure S5 — LysM -Cre induced effective Eng deletion in macrophages. Relative amount of the targeted Eng conditional 2fl allele in Eng2fl/2fl;LysM-Cre macrophages compared to that of Eng2fl/2fl macrophages. n = 3 per group. (TIF) [file pone.0088511.s006.tif]
